# Re-organisation of oesophago-gastric cancer care in England: progress and remaining challenges

**DOI:** 10.1186/1472-6963-9-204

**Published:** 2009-11-12

**Authors:** Thomas R Palser, David A Cromwell, Richard H Hardwick, Stuart A Riley, Kimberley Greenaway, William Allum, Jan HP van der Meulen

**Affiliations:** 1 Clinical Effectiveness Unit, The Royal College of Surgeons of England, 35-43 Lincoln's Inn Fields, London WC2A 3PE, UK; 2 Health Services Research Unit, Department of Public Health & Policy, London School of Tropical Medicine and Hygiene, Keppel St, London WC1E 7HT, UK; 3 Cambridge Oesophago-Gastric Centre, Addenbrooke's Hospital, Cambridge University Hospitals NHS Foundation Trust, Hills Road, Cambridge CB2 2QQ, UK; 4 Department of Gastroenterology, Northern General Hospital, Herries Road, Sheffield, South Yorkshire S5 7AU, UK; 5 National Clinical Audit Support Program, NHS Information Centre for Health and Social Care, 1 Trevelyan Square, Boar Lane, Leeds, LS1 6AE, UK; 6 Department of Surgery, The Royal Marsden NHS Foundation Trust, Fulham Road, London SW3 6JJ, UK

## Abstract

**Background:**

Oesophago-gastric cancer services in England have been extensively reorganised since 2001 to deliver a centralised, specialist-led service. Our aim was to assess how well the National Health Service (NHS) in England met organisational standards for oesophago-gastric cancer care.

**Methods:**

Questionnaires that asked about the provision of staging investigations, curative and palliative treatments and key personnel were sent in September 2007 to the lead clinician for oesophago-gastric cancer at all 30 cancer networks and 156 NHS acute trusts in England.

**Results:**

Responses were received from all networks and 81% of NHS trusts. All networks provided essential staging investigations and a range of endoscopic palliative therapies. Only 16 of the 30 cancer networks discussed all patients at the specialist multi-disciplinary team meeting and 11 networks had not fully centralised curative surgery. There was also variation between NHS trusts in the integration of the palliative care team, the availability of nurse specialists and the use of dieticians to provide nutritional support.

**Conclusion:**

There has been considerable progress in reforming oesophago-gastric cancer services but the process of reorganisation is still incomplete and regional differences in service provision exist that may lead to variation in patient outcomes.

## Background

Oesophageal and gastric cancer together represent the third most common cause of cancer death in the United Kingdom [1-3]. The prognosis is often poor with overall 5-year survival rates in the United Kingdom being approximately 8% for oesophageal cancer and 14% for gastric cancer [1,2]. The majority of patients present with advanced disease and many also have significant comorbidity and a poor level of overall fitness. As a result, the diseases' management, both curative and palliative, is complex and involves many different professional groups including surgeons, gastroenterologists, oncologists, palliative care physicians, radiologists, nurse specialists and dieticians [4-7].

During the 1990s, it was recognised that oesophago-gastric cancer services in England were fragmented and poorly organised. To overcome these weaknesses, the National Health Services (NHS) Cancer Plan [8] and the Improving Outcomes Guidance in Upper Gastro-Intestinal Cancer [5] set out a reform strategy and a number of specific recommendations. Firstly, all hospitals caring for cancer patients should be integrated into regional cancer networks. Within each network, curative services should be centralised into specialist cancer centres and a system should be established to coordinate care between these centres and other network hospitals (designated local units). Secondly, it was recommended that clinicians within each hospital should work together as a multi-disciplinary team (MDT).

The implementation of this reform strategy has seen oesophago-gastric cancer services undergo an extensive reorganisation. In this paper, we describe the results of a survey of English cancer networks and NHS acute trusts carried out in September 2007 that aimed to investigate the extent to which the reorganisation of oesophago-gastric cancer services had been completed, and to examine variation in the provision of diagnostic, therapeutic and support services. We also discuss these findings with the results of the Cancer Peer Review Programme carried out by the UK Department of Health between 2004 and 2007. The survey was performed as part of the National Oesophago-Gastric Cancer Audit, details of which can be found in its first annual report [9].

## Methods

Data on the organisation of oesophago-gastric cancer services were collected using two different questionnaires. The first contained questions that focused on the organisation of services within the cancer networks [see Additional file 1]. Networks are responsible for developing and planning all aspects of NHS cancer services within their region, including the process of centralisation. This requires coordinating the allocation of resources and establishing referral pathways between NHS acute trusts as well as other providers. The second questionnaire contained questions about the individual characteristics of services within the individual NHS trusts, focussing on the availability of key health professionals and the delivery of specific aspects of care [see Additional file 2]. Questions in both questionnaires were based on the recommendations in UK guidelines on the organisation of oesophago-gastric cancer services [5,6,10,11] (Table 1) and covered issues previously identified as essential for high-quality care [9].

**Table 1:** Published standards on which the survey questionnaires were based

*Network questionnaire*	*Trust questionnaire*
1. Treatment for patients with oesophageal cancer should be the responsibility of Specialist Oesophago-gastric Cancer Teams based in Cancer Units or Cancer Centres which would normally serve populations of at least one million. (IOG, p45)	1. The specialist palliative care team should be multi-professional, and should, as a minimum, include a palliative care physician and palliative care nurse specialists. (IOG, p61)
2. There should be 24-hour on-call consultant specialist surgical cover for postoperative care. Note: To achieve this measure at least 3 specialist consultant surgeons per team would be needed. (MCS, measure 2F-227)	2. A palliative care specialist should be a member of the Specialist Oesophago-Gastric Cancer Team and the Local Upper Gastro-intestinal Cancer Care Team. (IOG, p29-31)
3. The stage and spread of the cancer should be assessed using computed tomography (CT) or magnetic resonance scanning. If the patient is sufficiently fit to undergo radical treatment and imaging produces no evidence of widespread or metastatic disease, endoscopic ultrasound (EUS) should be used to estimate the depth of tumour penetration. If this also suggests that radical treatment could be successful, patients whose tumours could involve the peritoneal cavity should proceed to laparoscopy. (IOG, p37)	3. From the time of assessment, each patient should have access to a named clinical nurse specialist who can offer support and continuity of care. (IOG, p32)
4. Laser or photodynamic therapy should be used for initial control of obstructive symptoms caused by exophytic tumours in the oesophagus. Partially covered self-expanding metal stents should be used to control obstructive oesophageal symptoms either following or instead of laser therapy, depending on the availability of local expertise. (SIGN, p33-35)	4. Specialist advice should be available from a dietician. This should focus on helping patients to achieve adequate nutrition. Patients who have undergone surgery for oesophageal or gastric cancer should be given guidance to help them deal with post-surgical syndromes which can cause problems with eating. (IOG, p21-22)
5. Palliative chemotherapy should start within 2 weeks and ideally within 48 hours, depending on symptom severity. Chemotherapy with curative or adjuvant intent should start within 3 weeks and ideally within 1 week. Urgent radiotherapy, e.g., for spinal cord compression or superior vena cava obstruction, should start within 24 hours of referral. Palliative radiotherapy should start within 2 weeks and ideally within 48 hours, depending on symptom severity. Radical radiotherapy should start within 4 weeks and ideally within 2 weeks. (RCR)	5. All patients should be screened using a validated screening tool to assess nutritional risk. (SIGN, p24)

KEY to references:

SIGN = The Scottish Intercollegiate Guidelines Network (2006) [10]; IOG = Department of Health (2001) [5]; MCS = Department of Health (2004) [6]; RCR = The Royal College of Radiologists (2003) [11]

A database of English cancer networks and NHS acute trusts was prepared by combining information from various sources, including the Cancer Services Collaborative Improvement Partnership and the National Clinical Audit Support Program. Each network and NHS trust was then contacted to confirm the name and position of the lead clinician for oesophago-gastric cancer. The questionnaires were sent to these lead clinicians in September 2007 and non-responders were followed up by email and telephone.

## Results

Responses were received from all 30 cancer networks and from 38 of the 44 specialist cancer centres (84%) and from 88 of the 112 local units (79%). The structure of the networks are summarised in Table 2.

**Table 2:** Organisation of NHS oesophago-gastric cancer services in England at the time of the survey

English region	Cancer network	Number of NHS acute trusts			Number of responses from NHS trusts
			
		Cancer Centres	Local Units	Total	
North	Lancashire and South Cumbria	1	3	4	4
North	Greater Manchester and Cheshire	3	9	12	11
North	Merseyside and Cheshire	2	7	9	6
North	Yorkshire	2	5	7	7
North	Humber and Yorkshire Coast	1	2	3	3
North	North of England	2	7	9	8
East Midlands	North Trent	2	3	5	5
East Midlands	Arden	1	2	3	3
East Midlands	Mid Trent	1	2	3	2
East Midlands	Derby/Burton	1	1	2	2
East Midlands	Leicestershire, Northamptonshire and Rutland	2	1	3	3
West Midlands	Pan Birmingham	2	2	4	3
West Midlands	3 Counties	1	2	3	3
West Midlands	Greater Midlands	2	3	5	5
East of England	Mount Vernon	2	1	3	2
East of England	Thames Valley	2	4	6	6
East of England	Anglia	2	7	9	5
East of England	Essex	1	4	5	2
London	West London	1	6	7	4
London	North London	1	5	6	2
London	North East London	2	3	5	5
London	South East London	1	5	6	4
London	South West London	1	4	5	3
South East	Central South Coast	2	5	7	5
South East	Surrey, West Sussex and Hampshire	1	3	4	4
South East	Sussex	1	2	3	2
South East	Kent and Medway	1	3	4	3
South West	Peninsula	1	4	5	5
South West	Dorset	1	2	3	3
South West	Avon, Somerset and Wiltshire	1	5	6	6

### Cancer networks

The centralisation of oesophago-gastric curative cancer surgery was reported as still ongoing in 11 of the 30 networks. In four of the eleven, this involved centralising the work of one remaining unit. Services still required major restructuring in the other seven networks, with the lead clinicians in four of these reporting that the timescale for completion of this work was unclear.

The cancer networks identified 59 acute trusts that performed curative surgery for oesophago-gastric cancer. Of these, 14 (24%) were local units and one network reported that some patients received surgery in a Welsh cancer centre. All of the 45 specialist cancer centres and seven local units performed both oesophageal and gastric surgery. The seven other local units performed gastric surgery only. There were 152 surgeons working at these 59 acute trusts; 137 (90%) were upper gastrointestinal surgeons and 15 (10%) were thoracic surgeons (who worked at ten of the centres). The number of surgeons working at individual trusts varied from one to five (Table 3). Seven local units had a single surgeon and 53% of centres had three surgeons or more. Visiting surgeons based at local units carried out curative surgery in 18 specialist cancer centres.

**Table 3:** Number of surgeons performing oesophago-gastric curative surgery within NHS acute trusts identified by the 30 English cancer networks as providing this service

	Number of surgeons per trust				
	1	2	3	4	5
Cancer centres (n = 45)	0 (0%)	21 (47%)	14 (31%)	3 (7%)	7 (16%)
Local units providing oesophageal and gastric surgery (n = 7)	2 (29%)	5 (71%)	0	0	0
Local units providing gastric surgery (n = 7)	5 (71%)	2 (29%)	0	0	0

In 16 of the 30 networks, all patients were discussed at a specialist MDT meeting (i.e. an MDT meeting in a specialist cancer centre), while in the remaining networks only patients who were felt to require specialist input were discussed at these specialist MDT meetings.

The core staging investigations (CT scan, endoscopic ultrasound (EUS), laparoscopy) were available in all 30 networks, but access to EUS fine needle aspiration, PET, and PET-CT scan was variable (Table 4). The networks differed in terms of whether all or selected patients underwent particular investigations. Where investigations were performed in selected cases, the selections were based on clinical considerations rather than on patients' geographical location within the network.

**Table 4:** Reported availability of staging investigations in the 30 English cancer networks

Investigation		Patients on whom the investigation is performed			
	Tumour site	In all patients	In selected patients	None	Missing values
CT scan	Oesophageal	28 (93%)	2 (7%)		
	Junctional	28 (93%)	2 (7%)		
	Gastric	28 (93%)	2 (7%)		
					
Endoscopic	Oesophageal	17 (57%)	13 (43%)		
Ultrasound (EUS)	Junctional	16 (53%)	14 (47%)		
	Gastric	3 (10%)	22 (73%)	5 (17%)	
					
Staging	Oesophageal	2 (7%)	25 (86%)	2 (7%)	1
Laparoscopy	Junctional	12 (41%)	17 (59%)		1
	Gastric	19 (66%)	10 (34%)		1
					
EUS Fine	Oesophageal	3 (10%)	20 (67%)	7 (20%)	
Needle	Junctional	3 (10%)	20 (67%)	7 (20%)	
Aspiration	Gastric	2 (7%)	17 (57%)	11 (37%)	
					
PET Scan	Oesophageal	10 (33%)	17 (57%)	3 (10%)	
	Junctional	8 (4%)	19 (63%)	3 (10%)	
	Gastric	2 (7%)	16 (53%)	11 (37%)	
					
PET-CT	Oesophageal	7 (23%)	21 (70%)	2 (7%)	
	Junctional	6 (20%)	22 (73%)	2 (7%)	
	Gastric	1 (3%)	19 (63%)	10 (33%)	

Palliative treatment in the form of endoluminal stents and argon beam coagulation was available in all 30 networks, laser ablation or photodynamic therapy in 18, and brachytherapy in 16. All but two networks could provide endoscopic palliative therapy within two weeks of the decision to treat. Similarly, access to chemotherapy (both palliative and curative) could be provided within the same timeframe by 27 networks and to radiotherapy by 25.

### NHS acute trusts

106 of the 126 responding trusts (84%) had palliative care teams that included both a palliative medicine consultant and a palliative nurse specialist. Integration of palliative care into the multidisciplinary process was variable, with no member of the palliative care team routinely attending the MDT meetings at 10 of the 38 responding specialist cancer centres (26%) and 26 of the 88 local units (30%).

The number of clinical nurse specialists (CNS) varied between trusts. All 38 responding cancer centres had at least one full-time or two part-time CNS and 18 (47%) had two or three full-time CNS. Local units had fewer nurse specialists. Of the 88 responding local units, 15 (17%) had only one part-time CNS and nine had none at all.

All 38 responding specialist cancer centres and 83 of the 88 local units (94%) were able to provide dietician support. However, trusts differed in the groups of patients for whom dietician support was available and in the method of nutritional assessment made before patients began their treatment (Table 5). In particular, 33 of the 126 responding trusts (26%) had no dietician support for their surgical inpatients and 41 (33%) did not perform any formal nutritional assessment before starting treatment.

**Table 5:** Provision of nutritional support to oesophago-gastric cancer patients by the 126 NHS acute trusts who responded to the questionnaire

	Cancer centres (n = 38)	Local units (n = 88)
*Patients who have access to a dietician for specialist nutritional advice*		
Surgical inpatients	28 (74%)	N/A
All other Oesophago-Gastric cancer inpatients	34 (89%)	75 (85%)
Outpatients	32 (84%)	72 (82%)
No specialist support available	0	5 (6%)

*Methods of formal nutritional assessment prior to treatment*		
No formal assessment	9 (24%)	32 (36%)
Dietician assessment	26 (68%)	43 (49%)
Formal screening instrument (e.g. MUST score, Nutritional Risk Index)	3 (8%)	13 (15%)

## Discussion

Since the publication of the Improving Outcomes Guidance [5], oesophago-gastric cancer services in England have made considerable progress in developing a centralised, specialist service. We found that, in September 2007, all NHS acute trusts were organised into cancer networks and patients in all networks had access to the core staging investigations as well as to a range of palliative treatments. Coinciding with this reorganisation, the overall survival of patients with oesophago-gastric cancer has improved, with 1-year survival increasing from 30% to 37% between 1998 and 2005 [9]. However, we found that some components of this reorganisation were not yet complete. The centralisation of curative surgery was still in progress in 11 of the 30 cancer networks. Fourteen local units were still performing curative surgery and in seven of these, this was being performed by a single surgeon. Also, in only 16 networks were all patients discussed at a specialist MDT meeting. Moreover, palliative care teams were not fully represented in about a quarter of the multidisciplinary teams and some local units did not have a clinical nurse specialist.

The response rate to our questionnaires was high which strengthens the representativeness of our findings. There are three limitations to the study. Firstly, this was a cross-sectional study so the results do not provide evidence of change over time. Secondly, respondents provided self-reported data and we cannot exclude "social desirability bias" given that the lead clinicians may have wanted to give favourable answers. However, the likely impact of this bias is small because most questions addressed facts rather than perceptions.

Thirdly, the study examined the facilities and policy of each network, not what actually happens to patients. For example, while the policy in each network recommended that all patients being considered for curative treatment should have a CT scan, the levels of compliance within networks were unknown. Routine activity data could be used to describe progress in centralisation, but more detailed evaluation of processes such as disease staging requires prospective data collection. The National Oesophago-Gastric Cancer Audit will assess the process and outcomes of the care received by patients with this cancer in England and Wales [9].

The results of this "snapshot" survey confirm and complement those of the National Cancer Peer Review Programme that included visits to all cancer networks in England between November 2004 and March 2007 [12]. The Peer Review programme reported that overall compliance with the upper gastro-intestinal performance measures was 70%; compliance with a selected number of measures is shown in Table 6. Our results are more specific because the Peer Review process examined upper-gastrointestinal (upper GI) cancer services, which grouped oesophago-gastric cancer together with liver, pancreatic and biliary tract cancer.

**Table 6:** Key findings of the 2004 - 2007 Peer Review Programme of Upper Gastrointestinal Cancer services in English NHS acute trusts [12].

Aspect of care	Peer Review results	
Referral pathways	55% of the networks had referral guidelines agreed for diagnostic referral to secondary care	49% of the networks had guidelines agreed for referrals from secondary to tertiary care.
Network structure	There were significant gaps across all cancer sites in provision of oncologists, pathologists, radiologists, palliative medicine consultants and clinical nurse specialists.	37% of networks had specialist surgical teams with a 24-hour on-call rota (i.e. contained a minimum of 3 surgeons). There was wide regional variation in this from 13% (East) to 60% (South).
MDT structure	Units and centres had established their core MDTs in almost 100% of networks. Cover arrangements for core members (in case of annual leave etc) were in place overall in 58% of centres and 44% of units.	46% of cancer centres and 33% of local units achieved the standard of core members attending half of the MDT meetings
Clinical Nurse Specialist provision	The number of clinical nurse specialists per MDT was 1.4 for centres and 0.85 for units.	There was no clinical nurse specialist cover in 14% of cancer centres and 31% of local units, problems with workload and cover were reported in more than 20 centres and 30 units.

Peer Review found that the performance of upper GI cancer services was often worse than for other cancer sites. For example, the overall compliance rate of 70% was below that of breast, colorectal, lung and gynaecological cancer (77%, 77%, 73% and 75%, respectively). Similarly, attendance at the MDT meetings was the worst for any cancer site except lung with the core MDT membership present at less than half of the meetings (46% for centres and 33% for units). This compares to breast (77%), colorectal (58%) and gynaecology (83% for centres and 45% for local units). As in our survey, workforce issues were flagged up, especially with respect to the provision of clinical nurse specialists and palliative care teams. Overall upper GI cancer had the worst provision of nurse specialists of any cancer site. Compared to other services involved in the care of all cancer sites (such as oncology, radiology, etc.), specialist palliative care had the lowest overall level of compliance on the performance measures.

The Peer Review Programme was undertaken over a three year period while networks were reorganising their services. The reorganisation was supposed to be complete by the end of 2007 and this may be one reason why Peer Review found variation in how well the networks complied with the IOG recommendations. Our survey builds on Peer Review because it took place shortly before the deadline for full reorganisation. It is concerning that we found significant variation between networks in their provision of services, and that many had still not completed their reorganisation. It is also concerning that a minority were unable to give a date for completion.

The variation in service provision that we observed has implications for patient care and highlights several areas where improvements should be made. Firstly, there is good evidence that postoperative mortality is lower [13,14] and long-term survival is higher [15-17] if curative surgery is performed by high-volume specialist surgical teams. Over a third of the cancer networks had not completed the centralisation of curative surgery and just under half of NHS trusts were not providing 24-hour specialist consultant surgical cover. Of particular concern are the seven local units were still providing curative surgery with just one surgeon. There is a high-risk of complications after curative surgery and, for high quality postoperative care, surgeons with specialist knowledge and experience need to be continuously available.

Secondly, the provision of palliative care services, specialist nurses and nutritional support was variable and in a proportion of NHS trusts was insufficient. Studies have shown that patients with oesophago-gastric cancer require intensive support due to their poor quality of life and high level of symptoms [18,19]. There is also evidence that shows that patients who are malnourished have reduced survival, a worse quality of life [20,21], lower rates of completion of oncology treatment [22,23] and a higher rate of postoperative complications [22,23]. Nutritional support has been shown to reduce the impact of malnutrition and improve many of these outcomes [24]. A quarter of specialist centres do not formally assess their patients' nutritional status (either by dietician assessment or by a validated screening tool) before starting treatment. Simple tests such as the Nutritional Risk Index can be rapidly carried out by any doctor or nurse in the outpatients department and if performed on every patient could have a meaningful impact on their care.

Thirdly, although overall access to invasive palliative techniques was good with rapid access to stents and argon ablation therapy being available in all networks, access to other modalities was poor. Patients had to wait longer than 2 weeks for palliative chemotherapy in three of the thirty networks and for palliative radiotherapy in five networks. Brachytherapy was only available in 16 networks despite the fact that, compared to endoluminal stents, research has shown it to have fewer side-effects and provide better palliation in patients with an expected survival of greater than three months [25].

Finally, the Improving Outcomes Guidance recommended that all patients should be discussed by the specialist MDT [5]. The survey found that this occurs in 16 networks (53%). However, each network was also recommended to develop clinical guidelines on the selection of patients for referral to the specialist team [5]. The Peer Review Programme observed that these guidelines were in place in 71% of networks [11]. Consequently, not all patients are necessarily receiving the benefit of specialist experience. Processes need to be established so that each patient, irrespective of stage of disease or comorbidity, is evaluated by the network specialist teams, either by formal referral to the specialist MDT meeting or by discussion with a member of the specialist MDT.

## Conclusion

Although considerable progress has been made in transforming oesophago-gastric cancer care into a specialist, multi-disciplinary service, further improvements are necessary. Action is required to rectify the deficits in the provision of surgery, palliative care, clinical nurse specialists and nutritional support. Some of this depends on the further allocation of scarce resources but some aspects, such as ensuring all patients are discussed with a member of the specialist multidisciplinary team and are screened for malnutrition, can be improved by relatively simple changes in practice.

## Competing interests

The authors declare that they have no competing interests.

## Authors' contributions

DC, RHH, SAR and JvdM conceived the study; TP and DC designed the survey and questionnaires, with revisions suggested by RHH, SAR, KG, WA and JvdM; TP and DC conducted the statistical analyses and wrote the manuscript; RHH, SAR, KG, WA and JvdM commented on drafts; all authors read and approved the final manuscript.

## Pre-publication history

The pre-publication history for this paper can be accessed here:

http://www.biomedcentral.com/1472-6963/9/204/prepub

## Supplementary Material

Additional file 1 **Cancer network questionnaire**. The survey questionnaire sent to cancer network lead clinicians and which contained questions related to the organisation of cancer services within the network. Click here for file

Additional file 2 **NHS trust questionnaire**. The survey questionnaire sent to the lead clinician at each NHS trust and which contained questions about their hospital services. Click here for file
