# Supplementary material for: Re-organisation of oesophago-gastric cancer care in England: progress and remaining challenges
Source: BMC Health Serv Res. 2009 Nov 12;9:204. doi: 10.1186/1472-6963-9-204 (PMC2779810; doi:10.1186/1472-6963-9-204)
Supplement: Additional file 1 — Cancer network questionnaire. The survey questionnaire sent to cancer network lead clinicians and which contained questions related to the organisation of cancer services within the network. [file 1472-6963-9-204-S1.doc]

This questionnaire forms part of the organisational survey of the National Oesophago-Gastric Cancer Audit (NOGCA). The aim of the Audit is to examine the standard of care received by patients with oesophago-gastric cancer in England and Wales. The main component of the Audit is a prospective study that will run between 1 October 2007 and 31 December 2008. The organisational survey aims to examine issues which cannot be covered by the prospective study, such as differences in the availability of various treatment facilities. The organisational survey will involve sending questionnaires to both the network O-G cancer leads and the O-G lead clinicians of individual trusts.

Your contribution to the organisational survey would be greatly appreciated. The survey requires a high response rate to ensure its findings are accurate. The results of this survey will be published in the Audit’s first Annual Report in early 2008 with our analysis of existing data sources and qualitative study. Together, these should highlight various areas of good performance as well as areas where improvement can be made. If you have any questions relating to the project, please do not hesitate to contact us (see contact details below).

**Data protection statement**

All the information provided on this questionnaire will be treated as confidential. Published reports will only contain aggregated results and will not refer to any individuals or individual organisations.

**Instructions**

Please complete all questions on the questionnaire and return it in the stamp addressed envelope provided.

If you have network-wide imaging guidelines, we would also be very grateful if you could send us a copy of these guidelines.

Thank you for your assistance.

Mr Richard Hardwick Dr Stuart Riley

Lead clinician, AUGIS Lead clinician, BSG

Contact: tpalser@rcseng.ac.uk (clinical research fellow)

kimberley.greenaway@ic.nhs.uk (IC project manager)

RCS England, registered charity no. 212808

Please enter the name of your network___________________________________________

**Section 1: Organisation of staging investigations**

**1.** Please indicate what facilities you have for staging patients diagnosed with O-G cancer in your network. Please also indicate in which patients the investigations are used. Please note

- This question applies ONLY to patients who are potentially fit enough for definitive / radical therapy
- The option “All” means all or almost all patients, i.e. with few exceptions
- The option “None” means that it is never or very rarely performed

| We expect that a **CT scan** is available for oesophago-gastric (O-G) cancer patients in your network. Is this correct? | |
| --- | --- |
| Yes  No | a) On which patients is this investigation performed?  Oesophageal cancer: All Selected None  Cancer of gastro-oesophageal junction: All Selected None  Gastric cancer : All Selected None  b) If performed on selected patients, on what basis is the selection made? (tick all that apply)  Clinical / tumour characteristics  Geographical proximity to trusts with CT-Scan |
| We expect that **Endoscopic Ultrasound (EUS)** is available for O-G cancer patients in your network? Is this correct? | |
| Yes  No | a) On which patients is this investigation performed?  Oesophageal cancer: All Selected None  Cancer of gastro-oesophageal junction: All Selected None  Gastric cancer : All Selected None  b) If performed on selected patients, on what basis is the selection made? (tick all that apply)  Clinical / tumour characteristics  Geographical proximity to trusts with EUS |
| Is **EUS fine needle aspiration (FNA)** available for O-G cancer patients in your network? | |
| Yes  No | a) On which patients is this investigation performed?  Oesophageal cancer: All Selected None  Cancer of gastro-oesophageal junction: All Selected None  Gastric cancer : All Selected None  b) If performed on selected patients, on what basis is the selection made? (tick all that apply)  Clinical / tumour characteristics  Geographical proximity to trusts with EUS FNA |
| Is a **PET scan** available for O-G cancer patients in your network? | |
| Yes  No | a) On which patients is this investigation performed?  Oesophageal cancer: All Selected None  Cancer of gastro-oesophageal junction: All Selected None  Gastric cancer : All Selected None  b) If performed on selected patients, on what basis is the selection made? (tick all that apply)  Clinical / tumour characteristics  Geographical proximity to trusts with PET scan |
| Is a **PET-CT scan** available for O-G cancer patients in your network? | |
| Yes  No | a) On which patients is this investigation performed?  Oesophageal cancer: All Selected None  Cancer of gastro-oesophageal junction: All Selected None  Gastric cancer : All Selected None  b) If performed on selected patients, on what basis is the selection made? (tick all that apply)  Clinical / tumour characteristics  Geographical proximity to trust(s) with PET-CT scan |
| When **staging laparoscopy** is used: | |
|  | a) At which O-G cancer units is staging laparoscopy performed? (tick all that apply)  Specialist centres Local cancer units  b) On which patients is this investigation performed?  Oesophageal cancer: All Selected None  Cancer of gastro-oesophageal junction: All Selected None  Gastric cancer : All Selected None  c) If performed on selected patients, on what basis is the selection made? (tick all that apply)  Clinical / tumour characteristics  Geographical proximity to trust(s) performing laparoscopy |

**2**. Have network-wide imaging guidelines been produced for your network? Yes No

If Yes, please enclose a copy of the imaging guidelines document when returning the questionnaire.

**3.** If PET or PET-CT scans are not available for O-G cancer patients in your network, why is this?

____________________________________________________________________________________

____________________________________________________________________________________

**4.** Do some patients undergo part of their investigation or treatment outside the network?

Yes No

If No, please go to **question 5**

**4a.** Which patients are referred outside the network? (tick all that apply)

Patients undergoing specialist investigations

Patients undergoing chemotherapy

Patients undergoing radiotherapy

Patients undergoing surgery

**Section 2: MDT meetings**

**5.** Are all O-G patients in your network discussed at the specialist O-G centre(s)? Yes No

If not, what types of patients are discussed at the specialist centre MDT meetings? (tick all that apply)

Those patients needing specialist tests available on at the specialist centre

Those patients thought to be suitable for a curative treatment

Those patients who need specialist input into their palliation

**Section 3: Access to treatment facilities**

**Surgery**

**6a**. In the table below, please name the trusts in your network which perform surgical resections for oesophago-gastric cancer. Please also answer the questions on what type of surgery is performed and by which surgeons

| **Trust name** | **Does the trust perform:** | | **Does the trust have visiting surgeons from local cancer units?**  **(Tick all that apply)** | **How many surgeons perform resection procedures at this trust?** |
| --- | --- | --- | --- | --- |
|  | Gastric resections? | Oesophageal resections? |  |  |
|  | Yes  No | Yes  No | For gastric surgery  For oeso. surgery | Employed at the trust:  Upper GI surgeons _______  Thoracic surgeons _______  Visiting surgeons _______ |
|  | Yes  No | Yes  No | For gastric surgery  For oeso. surgery | Employed at the trust:  Upper GI surgeons _______  Thoracic surgeons _______  Visiting surgeons _______ |
|  | Yes  No | Yes  No | For gastric surgery  For oeso. surgery | Employed at the trust:  Upper GI surgeons _______  Thoracic surgeons _______  Visiting surgeons _______ |

**6b**. In the table below, please name the trusts in your network which do not perform surgical resections for oesophago-gastric cancer and the specialist centre to which they refer their patients. Include any specialist centre that is outside of your network.

| **Trust name** | **Specialist centres to which patients are referred**  **(Abbreviations of centre names can be used)** |
| --- | --- |
|  |  |
|  |  |
|  |  |
|  |  |
|  |  |
|  |  |

**Endoscopic Palliative Therapy**

**7**. What types of endoscopic procedure can be performed at specialist and local units in your network?

(tick all that apply)

a. Endoscopic stent insertion Specialist centres Local units

b. Laser ablation Specialist centres Local units

c. Photodynamic therapy Specialist centres Local units

d. Argon beam coagulation Specialist centres Local units

e. Brachytherapy Specialist centres Local units

**8**. Do any types of patients have difficulty in accessing endoscopic palliative therapy (within 2 weeks of the decision to treat)?

Yes No

If No, please go to **question 9**

8a. Which patients are affected? (tick all that apply)

Patients treated at local units

Patients treated at the specialist centre(s)

Patients referred to the specialist centre(s) from local units

8b. What are the reasons for these problems? (tick all that apply)

Lack of endoscopists

Lack of endoscopic equipment

Other reasons, please specify_______________________________________

**Oncological therapy**

**9.** Do any types of patients have difficulty in accessing oncological therapy (within 2 weeks of the decision to treat)?

Yes No

If No, please go to **question 10**

9a. Which patients have difficulties? Curative Palliative All

9b. For which types of therapy do difficulties in access arise? (tick all that apply)

Chemotherapy At specialist centres At local units

Radiotherapy At specialist centres At local units

**Section 4: Network structure**

**10.** Is the process of centralising tumour resective surgery now complete in your network?

Yes No

If no, please outline below the proposed changes in the space below.

Thank you for completing the questionnaire.

Please return to: Mr Tom Palser

Clinical Effectiveness Unit

Royal College of Surgeons of England

35-43 Lincoln’s Inn Fields

LONDON WC2A 3PE
