# Supplementary material for: Re-organisation of oesophago-gastric cancer care in England: progress and remaining challenges
Source: BMC Health Serv Res. 2009 Nov 12;9:204. doi: 10.1186/1472-6963-9-204 (PMC2779810; doi:10.1186/1472-6963-9-204)
Supplement: Additional file 2 — NHS trust questionnaire. The survey questionnaire sent to the lead clinician at each NHS trust and which contained questions about their hospital services. [file 1472-6963-9-204-S2.doc]

This questionnaire forms part of the organisational survey of the National Oesophago-Gastric Cancer Audit (NOGCA). The aim of the Audit is to examine the standard of care received by patients with oesophago-gastric (O-G) cancer in England and Wales. The main component of the Audit is a prospective study that will run between 1 October 2007 and 31 December 2008. The organisational survey aims to examine issues which cannot be covered by the prospective study, such as differences in the availability of various treatment facilities. The organisational survey will involve sending questionnaires to both the network O-G cancer leads and the O-G lead clinicians of individual trusts.

Your contribution to the organisational survey would be greatly appreciated. The survey requires a high response rate to ensure its findings are accurate. The results of this survey will be published in the Audit’s first Annual Report in early 2008 with our analysis of existing data sources and qualitative study. Together, these should highlight various areas of good performance as well as areas where improvement can be made. If you have any questions relating to the project, please do not hesitate to contact us (see contact details below).

**Data protection statement**

All the information provided on this questionnaire will be treated as confidential. Published reports will only contain aggregated results and will not refer to any individuals or individual organisations.

**Instructions**

Please complete all relevant questions on the questionnaire and return it in the stamp addressed envelope provided. Some questions relate only to specialist O-G cancers centres, while others relate to local units only.

Thank you for your assistance.

Mr Richard Hardwick Dr Stuart Riley

Lead clinician, AUGIS Lead clinician, BSG

Contact: tpalser@rcseng.ac.uk (clinical research fellow)

kimberley.greenaway@ic.nhs.uk (IC project manager)

RCS England, registered charity no. 212808

Please enter your trust name: ___________________________________

**Section 1: Access to palliative care and nutritional support**

**1.** Which clinicians constitute your palliative care team? (tick all that apply)

1. Consultant in palliative medicine
2. Specialist Nurse in palliative care
3. Other staff, please specify ___________________________________

**2.** Which members of your palliative care team routinely attend the Oesophago-Gastric (O-G) cancer MDT meetings? (tick all that apply)

1. Consultant in palliative medicine
2. Specialist Nurse in palliative care
3. Other staff, please specify ___________________________________

**3.** At your trust, which patients with O-G cancer have access to specialist nutritional advice from a dietician? (tick all that apply)

1. Surgical inpatients only
2. All other O-G cancer inpatients
3. O-G cancer patients when seen as an outpatient

**4**. Typically, at your trust, how is the nutritional status of O-G cancer patients formally assessed prior to treatment (such as surgery or chemotherapy)?

1. There is no formal assessment prior to treatment
2. Dietician assessment
3. With a standard screening instrument, eg. Nutritional Risk Index (NRI)
4. Using a locally-developed screening instrument
5. Other method, please specify ___________________________________

**Section 2: Specialist Nurse provision**

**5**. How many specialist nurses for O-G cancer are employed at your hospital (excluding nurse endoscopists)?

Full-time _______________

Part-time_______________ (include nurses shared with other specialties here)

**Section 3: Quality of Life assessment**

**6**. In which patients with O-G cancer does your hospital routinely measure quality of life?

Tick all that apply, and, where applicable, please state the instrument used.

1. Patients undergoing curative treatment *Instrument* ______________________________
2. Palliative patients *Instrument* ______________________________
3. Quality of life is not routinely assessed

**7**. If Quality of Life is routinely measured, how are the measurements used in clinical practice?

(tick all that apply)

1. To aid treatment selection
2. To monitor the outcomes of treatment
3. Other, please specify ___________________________________________
4. Not applicable

**Section 4: Patient information**

**8**. Does your hospital provide written information regarding the following? (tick all that apply)

1. Information about the disease, diagnostic procedures and the

options for treatment

1. Information about diet and minimising problems with eating
2. Information relating to local or national patient support groups
3. Other information, please specify________________________________________

**Section 5: Organisation of MDT meetings**

**9**. Does your trust act as a specialist O-G cancer centre within your network?

Yes If yes, go to question 12

No

**10**. Do you have combined (eg. video-linked) MDT meetings with the specialist O-G cancer centre?

Yes No

**11**. Which patients are referred to the specialist O-G cancer centre? (tick all that apply)

Those patients needing specialist tests available on at the specialist centre

Those patients thought to be suitable for a curative treatment

Those patients who need specialist input into their palliation

**12**. What issues are reported and discussed at your MDT meetings? Include the topics at combined unit –specialist MDT meetings (tick all that apply)

1. Decision about the appropriate treatment plan
2. Changes to a previous treatment plan
3. Results of curative surgery (including pathology
4. Results of definitive (radical) oncological therapy
5. Other, please specify__________________________________________________

**13**. Which of the following staff support your MDT meetings? (tick all that apply)

1. MDT coordinator
2. A data clerk (responsible for collecting clinical data)

**14**. Is patient information presented at the MDT meeting entered into an electronic IT system (either at the meeting or shortly afterwards)? Yes No

**Section 6: Assessment of fitness for surgery**

**(To be completed by specialist O-G centres only)**

**15.** What investigations do you routinely perform for assessing fitness for surgery (excluding routine blood tests)? Please note this refers to the routine tests performed on all / most patients, not those only performed in patients with higher degrees of comorbidity.

Oesophageal resections (tick all that apply):

1. Pulmonary Function Tests
2. Arterial blood gas analysis
3. Echocardiogram
4. Cardiac stress test (including stress echocardiogram, treadmill test, perfusion scan)

Gastric resections

1. Pulmonary Function Tests
2. Arterial blood gas analysis
3. Echocardiogram
4. Cardiac stress test (including stress echocardiogram, treadmill test, perfusion scan)

Thank you for completing the questionnaire.

Please return to: Mr Tom Palser

Clinical Effectiveness Unit

Royal College of Surgeons of England

35-43 Lincoln’s Inn Fields

LONDON WC2A 3PE
